# Curbing antimicrobial resistance in post‐COVID Africa: Challenges, actions and recommendations

**DOI:** 10.1002/hsr2.771

**Published:** 2022-08-08

**Authors:** Deborah Oluwaseun Shomuyiwa, Don Eliseo Lucero‐Prisno, Emery Manirambona, Mohamed Hoosen Suleman, Rehab A. Rayan, Junjie Huang, Thaint Nadi Zaw, Yusuf Babatunde, Salomey Asaah Denkyira, Shuaibu Saidu Musa

**Affiliations:** ^1^ Faculty of Pharmacy University of Lagos Lagos Nigeria; ^2^ Department of Global Health and Development London School of Hygiene and Tropical Medicine London UK; ^3^ Faculty of Management and Development Studies University of the Philippines, Open University Los Baños Laguna Philippines; ^4^ Faculty of Public Health Mahidol University Bangkok Thailand; ^5^ College of Medicine and Health Sciences University of Rwanda, Kigali, Rwanda Kigali Rwanda; ^6^ Nelson R. Mandela School of Medicine University of KwaZulu‐Natal Durban South Africa; ^7^ Centre for the AIDS Programme of Research in South Africa Durban South Africa; ^8^ Department of Epidemiology, High institute of Public Health Alexandria University Alexandria Egypt; ^9^ JC School of Public Health and Primary Care, Faculty of Medicine The Chinese University of Hong Kong Hong Kong PR China; ^10^ Oxford University Hospitals NHS Foundation Trust Oxford United Kingdom; ^11^ Faculty of Pharmaceutical Sciences University of Ilorin Ilorin Nigeria; ^12^ Faculty of Pharmacy and Pharmaceutical Sciences Kwame Nkrumah University of Science and Technology Kumasi Ghana; ^13^ Department of Nursing Sciences Ahmadu Bello University Zaria Nigeria; ^14^ Global Health Focus Africa Abuja Nigeria

**Keywords:** Africa, anti‐infective agents, antimicrobial resistance, COVID‐19, drug resistance, infection control

## Abstract

**Background:**

Antimicrobial self‐medication and use have significantly increased in the COVID‐19 era—increasing antibiotic consumption and resulting in a high prevalence of antimicrobial resistance in Africa (AMR). We conducted a narrative review to investigate challenges associated with curbing AMR in a post‐COVID‐19 setting in Africa, suggesting practical measures applicable for policy‐informed implementation.

**Method:**

A narrative review was performed to pinpoint AMR challenges and actions on the African continent. A comprehensive search was conducted in the scientific databases that include PubMed, PubMed Central and Google Scholar using predetermined search terms.

**Results:**

The emergence of the COVID‐19 outbreak has added to the challenges of tackling AMR on the continent, which has jeopardized AMR interventions' hard‐won gains. Identified challenges have been Health systems disruption, Irrational Antimicrobial Use, Weak Antimicrobials Regulatory Ecosystem, Inefficient Population Infection Prevention, and Control Practices, Inadequate access to Health Services and data challenge on AMR surveillance.

**Conclusion:**

The COVID‐19 pandemic fueled AMR in Africa. There is a need for AMR control post‐COVID, such as measures for ongoing antimicrobial stewardship and good infection control practices. Further, curbing AMR requires rigorous regulatory enforcement and efficient AMR Surveillance. There should be a body to raise AMR awareness among the population. Research, Innovation and Technology could play an essential role supported by capacity building and global partnership.

## 1 INTRODUCTION

The coronavirus disease 2019 (COVID‐19) pandemic has significantly impacted Africa, with the vulnerable population bearing the brunt of the burden.^1^ The fear of mortality due to the pandemic has been high,^2^ while health care services focus on the pandemic. The availability of staff and accessible quality medications play an essential role in responding to the outbreak and are considered critical bodies of health infrastructure.^3^ On the contrary, the understaffing and the lack of drug supply pose a challenge to the health systems, thus impelling poor health outcomes.^3^

As African health systems were inadequately prepared to respond to the pandemic, the adverse effects on health services have been numerous. There was a lack of accessible quality health care services, compounded by the COVID‐19 surge and paucity of equipment such as testing material. Similarly, the protective policies devised to curb the pandemic have decreased the seeking for health care, and some already‐scheduled services have been canceled or missed.^4^ These factors have contributed to self‐medication and inappropriate antimicrobial use.^5^ Self‐medication is associated with the emergence of antimicrobial resistance (AMR).

During the COVID‐19 pandemic, self‐medication has extensively increased in Africa.^6^, ^7^ Self‐medicated drugs have included but were not limited to antibiotics such as Metronidazole, Amoxicillin, Azithromycin, Ciprofloxacin, and antimalarial including Hydroxychloroquine and Chloroquine.^6^ An increased antibiotics self‐medication in uncomplicated illnesses in the population in resource‐constrained Africa is a growing issue, leading to a high prevalence of AMR.

AMR has been one of humanity's most significant global health threats today. It is a public health problem that transcends continents and national borders and is rapidly growing, as seen with the sixfold increase in resistance rates since 2017.^8^ For instance, antimicrobial‐resistant bacterial strains could lead to untreatable infections and cause 10 million deaths each year by 2050.^9^ Understanding AMR in Africa post‐COVID‐19 can prevent its extension and avoidable deaths. However, little is known about AMR in the post‐COVID‐19 context in Africa. There is a need to conduct research to provide strategies and policy responses for a resilient recovery post‐COVID, essential in driving a systems approach to ensuring population safety.^10^ This narrative review explores challenges related to curbing AMR in a post‐COVID‐19 setting in Africa and provides recommendations for practical measures useful for policy‐informed implementation.

## 2 METHOD

We conducted a narrative review of data sources to identify AMR challenges and actions in African countries. A comprehensive search on PubMed, PubMed Central and Google Scholar was conducted using predetermined search terms. The inclusion criteria were data sources, that is, relevant articles about AMR interventions and associated successes and challenges in African countries and were published in English. References of the data sources were also reviewed to identify relevant information. Supporting Information Data were also gathered from country reports, commentaries, policy briefs and other reports. The collected articles were managed using Mendeley Reference Manager. The extracted data were discussed narratively to explore the aim of the study.

## 3 RESULTS/DISCUSSION

### 3.1 Burden of AMR

The distribution of substandard and counterfeit antimicrobials has a significant contribution to the spread of AMR, particularly in Africa.^11^ Before the COVID‐19 outbreak, with over 700,000 deaths annually,^12^ AMR was a pandemic, circumventing health care delivery globally, claiming 1.27 million deaths worldwide and the six leading resistant pathogens associated with over 92 million deaths as of 2019.^13^ In Africa, the significant impact of AMR is becoming more apparent, with western sub‐Saharan Africa having the highest all‐age death rate of 27.3 deaths per 100,000.^13^ This concern is compounded by a weak and fragmented public health system across the continent and a high burden of infectious diseases, with 62% of disability‐adjusted life years (DALYs) in the African region attributable to infectious diseases.^14^ With COVID‐19 ruling most aspects of health care delivery globally, the effects of this phenomenon are gaining more impact. The rising and evolving public health concern is associated with increased morbidity and mortality.^15^

### 3.2 Global Action Plan (GAP) and implementation in Africa

Compounded by COVID‐19, AMR can drive more people into poverty, jeopardize global health security, and obstruct progress toward the Sustainable Development Goals and universal health care.^16^ Africa is already at a loss, with more than half of the world's poorest communities coupled with the high burden of infectious diseases. The design of the GAP on AMR is to align the optimal use of antimicrobials and to develop the knowledge and evidence base through research and surveillance.^17^ This GAP provided a template for countries to create their AMR national action plans (NAPs). Using an incremental approach lays out the crucial steps that the various stakeholders should take to address AMR over the next 5−10 years. However, Africa is slow on the uptake in aligning with current international efforts to fight the increasing pandemic of AMR as few countries have AMR NAPs. Challenges that include a mix of insufficient local awareness, inadequate data and knowledge of AMR impact, and a lack of technical and financial resources for implementation and research and progress monitoring have stalled the action plans.^18^, ^19^

Implementing the NAPs is very cost‐intensive, a luxury Africa cannot afford. For instance, it was estimated that the implementation of Zimbabwe's NAP would cost $44.6 million over 5 years from 2017.^20^ Interestingly, some countries have even estimated double that amount for their implementation. The World Health Organization (WHO) insisted that African countries must raise health budget expenditures if they wish to enhance their people's health and meet international health targets.^21^ AMR can escalate the cost of health care, and most African countries have failed to meet their Abuja Declaration commitments of setting aside 15% of their national budget for health. A governance framework is required to guide and support researchers and stakeholders in developing and assessing these NAPs.^22^

### 3.3 Challenges of AMR control in Africa

The emergence of the COVID‐19 outbreak has elevated the stakes for AMR response. Health systems responses and public health priorities have evolved due to the pandemic, and this evolution has threatened the hard‐won gains of AMR interventions (Table 1).

**Table 1:** Challenges of AMR control in Africa

1. Weak and inadequate health care delivery.
2. Irrational antimicrobial use enabled by unrestricted or unmodulated access to antimicrobials. Prolonged empiric use of antimicrobials and health systems inadequacies are enabling irrational prescribing.
3. Weak regulatory capacity and logistic gaps result in fractious access and an upsurge in the circulation of counterfeit/substandard medicines.
4. Inadequate Antimicrobial surveillance data.
5. Shunted access to health care.
6. Poor hygiene and inadequate infrastructure for infection prevention.

#### 3.3.1 Health systems disruption

While most African health systems adopt a hierarchical design in health care provision, lower‐tier levels with community proximity are underfunded and underutilised, limiting the health system's capacity. The weak health system, inadequate health care delivery and shortages of supply of essential medicines in public facilities limit the ability and capacity to tackle AMR.^23^ While the development of AMR has been attributed to the increasing antimicrobial utilization, poor governance, corruption, and lack of adequate control due to fragmented health systems management are important drivers of the epidemic. A lapse in oversight and enforcement of policies and regulations relating to antimicrobials distribution furthers the plight.^24^ The lack of specific funding sources, especially budgetary allocation, is a challenge as the only official allocation for the federal budget for AMR activities was found in Nigeria.^24^

The COVID‐19 pandemic has had a significant impact on health systems. Effects range from the deprioritisation of AMR presented as funding challenges for AMR partnerships and bias in the collection of AMR data with admissions and procedures influenced by the pandemic, health workforce shortage, workforce and health system resources in the pandemic response.^5^ COVID‐19 has had a sweeping effect on new and existing AMR partnerships and stalled the oversight and accountability of AMR interventions.^25^

#### 3.3.2 Irrational antimicrobial use

Human behavior has been the primary driver of AMR, and the problem of nonprescription antimicrobial consumption is widespread in Africa. Widespread and indiscriminate empirical use of antibiotics has been a significant risk factor for AMR development in Africa. AMR emergence is fostered in environments where it is common practice to buy antibiotics over the counter. In developing environments, excessive use is attributed to ease of access and perception of antibiotics as “wonder drugs,” while amongst the affluent, inappropriate indication plays an important role.^26^ In Sub‐Saharan Africa, over 70% of antibiotics are supplied without prescriptions.^27^

Around 70% of COVID‐19 inpatient or outpatient settings received antimicrobials primarily for empiric use.^28^ It has been discovered that broad‐spectrum antibiotics were mainly recommended for mild to moderate COVID‐19 management, which violates WHO guidelines and significantly reduces selection pressure.^29^ Without much evidence to support its use, Azithromycin is common in treating COVID‐19 patients globally, including in Africa.^30^ The high workload and stress worsened by the COVID‐19 pandemic give little room for adequate therapy and medication review.

#### 3.3.3 Weak antimicrobials regulatory ecosystem

In Africa, where low and middle‐income countries predominate, antimicrobial purchasing is less regulated.^29^ Over‐the‐counter antibiotics procurement is common in Africa, as 100% of antibiotics can be procured without prescriptions in some African countries.^27^ Antimicrobials can be procured on the roadside, in small shops and stores, dispensed by auxiliary nurses in communities, and irrationally dispensed by pharmacies who put economic interest over the public good.^31^ The weak enforcement of regulations promotes irrational use of antimicrobials, resulting in AMR. Weak regulatory systems and logistic deficits can cause the circulation of substandard/counterfeit medicines and procurement gaps that foster inadequacy in health systems' drug regulatory capacity. A strong antibiotics regulatory capacity can push toward developing new antibiotics that can respond to resistant strains. Notwithstanding that, the lack of a robust antibiotics regulation resulted in zero new antibiotic development in the last 30 years ago; a phenomenon described as the “discovery void.”^32^ This can be explained by the fact that the development of new antibiotics is not very beneficial to drug development companies, which prefer more profitable drug development.

#### 3.3.4 Inefficient population infection prevention and control (IPC) practices

IPC remains the core of fighting against AMR. IPC seeks to improve hygiene, waste disposal and infrastructure. The failure to devise and comply with IPC leads to the rise of AMR. For instance, water, sanitation, and hygiene (WASH) have been described as essential for AMR in Kenyan health care settings, with poor WASH deemed to increase AMR.^33^ Poor hygiene may also lead to an avoidable prescription of antibiotics for cases that a proper WASH can simply handle.

Furthermore, better infrastructures are critical to better IPC. The poor infrastructures can lead to a significant increase in infections and AMR. Notably, an international study investigating the quality of health care facilities in 78 Lower‐ and Middle‐Income Countries (LMICs) reported poor outcomes. Overall, 39% of health care facilities had no handwashing soap, 33% had no improved toilets, 59% lacked reliable electricity, 50% had no piped water, and 39% of health care care facilities had inadequate infectious waste disposal.^34^ Those factors show that there are poor health care facilities in LMICs, thus suggesting poor hygiene and sanitation and, as a result, the spread of resistant microorganisms to antibiotics.

#### 3.3.5 Inadequate access to health services

Despite the considerable improvement in global health, millions of people still lack access to quality health services, including effective antimicrobial medicines.^35^ A primary driver of AMR is the misuse and abuse of antimicrobial drugs. Long distances, poor or nonexistent road infrastructure, public transportation, or seasonal weather conditions can exacerbate accessibility problems.^36^ Retail shops tend to be nearer to patients and clients than other institutional health facilities, especially in rural areas, and often become the only source of care, hence the likely overuse or misuse of antimicrobials.^37^

#### 3.3.6 The data challenge on AMR surveillance

Managing Data is among the significant obstacles to AMR surveillance in Africa. Local stewardship is usually dependent on the availability of local antimicrobial susceptibility data. The Global Antimicrobial Resistance Surveillance System (GLASS) developed by WHO to support GAPs for AMR aims to foster standard AMR surveillance globally. While AMR reporting and surveillance have seen exponential growth globally from 2017 to 2019, reports indicate that few African countries have practical and functional surveillance systems.^38^ A systematic review enunciated that AMR data is nonexistent in over 40% of African countries,^39^ even with high determined resistance to commonly prescribed antibiotics. WHO ascertained that this data limitation in African countries is due to limited laboratory and diagnostic capacity and functional surveillance networks.^30^

The deficiency of trained personnel, confined expertize and experience in the discipline, deficiency of calibration of antimicrobials susceptibility testing, inadequate sampling of the case with suspected infection and many origins of data from the drug industry, international systems, hospital and private labs, and national surveillance channels cause disconnected and sporadic data. With AMR risk being highest in Sub‐Saharan Africa,^13^ data regarding antibiotics use and AMR surveillance systems are inadequate. The lack of systematic diversity and streamlining results in unrepresentative and unreliable data. Other elements are linked to the constricted or shortage in accessing technology that eases data production, exploration, and distribution. The COVID‐19 pandemic has effected a shift in infrastructure and procedures for reporting and information systems in AMR surveillance.^25^

## 4 RECOMMENDATIONS

To combat and win the fight against AMR, African countries need to drive and implement comprehensive plans and strategies. This is especially important as the COVID‐19 pandemic management has been dedicated to the majority of resources and workforce in recent times. Health care delivery is a complex adaptive process, and the AMR challenge requires adequate systemic adaptation to foster sustainable change. All stakeholders, policymakers, and health systems partners should be involved in providing a wholesome policy package to combat AMR in the complicated COVID‐19 system. Adopting a systems approach is crucial in the complex and adaptive system of people, processes, resources, and institutions. Africa needs to drive the implementation of sustainable interventions with support regarding resources, including funding, strategy, data utilization, and management and capacity merged with leadership, accountability, and transparency (Table 2).

**Table 2:** Recommendations

1. Awareness of the increasing risk of antimicrobial resistance and infectious disease management. The utilization of communication theories and social context to harness public understanding and audience engagement.
2. Encouraging Antimicrobial stewardship evidence‐based updates on practice guidelines of health professionals, routine and independent review of stewardship practices, and collaboration with medical and pharmaceutical professional bodies.
3. Improving access and quality of care by optimizing primary health care.
4. Improved AMR surveillance with a focus on antimicrobial utilization and changes in epidemiology. Collaboration to improve the quality of surveillance systems.
5. Assessment of progress and updates on National Action Plans for AMR. Accountability mechanisms and change management system frameworks for AMR policies and interventions.
6. Adequate funding, support and autonomy for regulatory enforcement procedures and the development of validated systems, practices and policies for quality systems. Unauthorized access to antimicrobials should be discouraged—enforcement of scope of practice of patent drug vendors.
7. Improving laboratory capacity and data collection systems.
8. Implementation of research with collaboration and community engagement at the core and innovation with technology for quality health.
9. AMR frameworks must strengthen the competence and capacity of the health workforce for health systems' resilience. Undergraduate training must incorporate AMR training into undergraduate studies to promote stewardship.
10. Global dialogues and advocacy. Collaboration with advocacy groups to promote continuity of intervention and optimize reach.

### 4.1 AMR awareness

In 2015, the GAP on AMR adopted by WHO listed improving awareness through effective communication, education, and training as its first objective.^40^ This is essential to driving human behavior toward AMR mitigation as risk communication facilitates audience engagement. Open communication between health care professionals, the public, government, media, and researchers was key in COVID‐19 management.^41^ Africa's policy agenda must be driven first and foremost by knowledge management. Harnessing public understanding of infectious diseases and AMR has a significant impact on encouraging efficient AMR transmission control techniques.^42^ AMR and its behavioral impact are unique. Communication theories and beliefs that drive behavioral change should be designed and implemented with proper evaluation to achieve sustainable change in the African context. Audience segmentation, public agenda setting, and AMR framing are all practices that can help with message development.^40^ AMR information and infectious diseases management education dissemination in local language harnessing primary health centers optimizes regional reach.

### 4.2 Antibiotic stewardship

AMR control and management require the adaptation of functional and sustainable antimicrobial stewardship measures. Rational prescribing should be promoted to advocate for stewardship. This advocacy will include developing evidence‐based guidelines for antimicrobial prescribing in private and public facilities,^25^ an independent review of the antimicrobial stewardship practices of professionals in these facilities, and the development of measures to limit poor prescribing practices. Diagnostic stewardship is essential for antimicrobial stewardship and adequate infection control. Good laboratory practices are essential in quality management and capacity.^43^

Collaboration with the medical and pharmaceutical professionals to ensure practice regulation and adherence to guidelines is essential. This collaboration includes rational prescribing, prescription monitoring/surveillance, patient education, formulary approval and audit, computerized decision support, and continuing medical training. Incentivising antimicrobial stewardship in LMICs health care sectors will create room for cooperation and coordination. Dedicated funding commitment and social support packages implemented by governments and stakeholders are required to drive successful interventions. Social drivers of antimicrobial practices should be incorporated and adequately explored in the policy framework for acceptable balance between access and antimicrobial stewardship.

Antibiotic stewardship policies should facilitate access to medicines and care and encourage seeking the primary health care practice. “Urban advantage” of proximity to quality health services, drugs and medical supplies for health maintenance needs to be translated in rural Africa.^44^ Primary health care can offer good service delivery with population infection control with funding and capacity development.

### 4.3 Efficient AMR surveillance

One of the five crucial objectives of the WHO GAP is to tone up data on AMR via research and surveillance. Aligning with the GLASS developed by WHO and creating local repositories of standard AMR data, analysis and distribution inform the decision making on a national and international scale.^30^ COVID‐19 and its associated changes, such as increased antimicrobial use, underline the importance of optimizing AMR surveillance to monitor AMR trends with global health changes.^25^ AMR data management should assess biases in current analyses of AMR surveillance data.

AMR surveillance in the wake of irrational antibiotic use in the context of the COVID‐19 pandemic is essential to adapt to changes in AMR epidemiology.^42^ Focus on changes in antimicrobial utilization, diagnostics and resistance reporting is vital. A study in Ethiopia demonstrated that the development of standard AMR surveillance is attainable with solid leadership and stakeholder engagement.^45^ Collaboration of African countries with GLASS to improve the quality of surveillance systems makes for reliable data of global capacity. The active dissemination of surveillance data amongst policymakers and stakeholders is vital to drive their use as evidence in policymaking—these work to provide relevant and scientifically valid epidemiological data to base decisions and policies on AMR.

### 4.4 Regulatory enforcement

Consistent regulatory systems that work do not just materialize; they require planning, design, and concerted implementation. At the same time, African governments need in‐depth analysis and assessment and updates on NAPs.^24^ Policies should promote success and delimit the gaps in implementation with best practices to mitigate future AMR threats. Regulatory interventions require collaboration amongst policymakers, influencers, and the implementation body. Organized scrutiny and accountability of governments to meet policy commitments and mandatory and comprehensive progress reports on the implementation of NAPs are vital to address the slow progress in the AMR change management system and eradicate the systematic communication challenge encountered in NAP implementation.^24^

Harmonization of law and regulations on drug manufacturing procurement, logistics and treatment practices is important. Regulatory authorities require adequate funding, strategic support, authorization and autonomy to undertake enforcement procedures, including food and veterinary systems.^46^ Stricter regulation and enforcement of policies that stifle nonprescription and irrational use of antibiotics are essential to driving stewardship. This is crucial to managing the antimicrobial utilization practices of the patent drug vendors in Africa. Adequate enforcement of the scope of practice of patent drug shops and specialized retail drug outlets is essential. The development and review of validated systems, practices, and procedures are crucial for quality systems.

### 4.5 Implementation of infection prevention policies

With the COVID‐19 management, health systems triggered infection prevention policies by appropriately isolating cases and public health practices, including personal protection.^37^ Continuous advocacy for IPC procedures, including appropriate handwashing, clean water and sanitation protocols and correct use of personal protective equipment, makes functional infection prevention.

Beyond the biological origins of AMR, social problems also drive perspectives on disease and infection interactions and management.^47^ Policymakers have to gain deeper understanding health care needs of the population and models of service delivery to promote infection control amongst the population. Fueling the primary health care practice is essential to facilitate access to medicines and quality health care. The primary health care cadre is pivotal to supporting health system resilience in Africa. More attention to funding, and capacity development, including training personnel and the populace on infection prevention measures.

The AMR plight in Africa highlights the value of creating policies for birthing IPC programs, amending access to necessary second‐line antibiotics as required, and producing novel vaccines and antibiotics. In addition, high‐quality data on communicable diseases, infectious agents, and AMR are merely available in several low‐income contexts. Preventing bacterial AMR and growing microbiological lab capability and data collection systems to better scientific interpreting of such humankind's well‐being danger should be an exceedingly great priority for worldwide health policymakers.

### 4.6 Research, innovation, and technology

Information management and innovation are at the center of AMR response. Studies on consumption practices and their consequences and national statistics on AMR and public health responses can help shape public policy, provide predictive evidence of AMR development, and serve as an essential evaluation tool. Adequate policy implementation research on AMR is critical for designing interventions in resource‐constrained settings of Africa.^48^ It provides knowledge, drive, and architecture to prepare for present and future challenges related to AMR emergence.^5^ Collaboration and community engagement with policymakers and relevant stakeholders should define the contributions of the AMR research community. Further studies investigating AMR evolution with the COVID‐19 pandemic and changes in consumption practices.

Africa's technology is rapidly growing. The COVID‐19 pandemic has brought to life the critical need for the rapid development of accurate diagnostic methods, vaccines and antimicrobial treatments to improve health outcomes, especially in the vulnerable population.^49^ Innovative measures utilizing technology can moderate the distribution of antimicrobials and promote antimicrobial stewardship by providing health information. Information technology is a powerful tool for health promotion and raising awareness.^50^ Rapid testing geared toward identifying susceptible antimicrobials can help judicious selection of antimicrobials. Directing innovation toward the control of antimicrobials and AMR surveillance is essential in the AMR fight.

### 4.7 Capacity building

The capacity for a responsive AMR health system requires adequate infrastructure and a competent health workforce. The COVID‐19 pandemic calls for reestablishing partnerships and training programs for AMR responses. Leveraging knowledge gained from the pandemic response makes for strengthening health systems. Continued education on patient management improves appreciation of clinical guidelines across medical competencies.^25^ A multidisciplinary approach is vital in developing health workforce capacity development and training. Risk limitation and maximization of impact of interventions should incorporate strategic competencies and specialities and not just prescribers and dispensers. Capacity in the health workforce for health systems resilience requires good management and leadership skills. Influence in practice stems from shared responsibility to the AMR cause. Health care must incorporate a review of procedures to qualify health care delivery and antibiotic delivery practices systemically into health systems. Integration of AMR training in undergraduate studies improves the students' appreciation of antimicrobial stewardship and practice quality.

### 4.8 Global partnerships

Global dialogues drive the synthesis of knowledge and experience that informs policy development. Harnessing advocacy and the potential of the virtual and remote space can drive the AMR mandate. Policy design, research advocacy, education advocacy, and community support and development represent several advocacy contexts. Partnerships should explore communication models that drive participation and shared community amongst decision‐makers and stakeholders. While COVID‐19 mortality in Africa has been lower, the rapid spread of the virus is a call to strengthen response capacity and create a quality standard for interventions.^30^ Collaboration between governments and advocacy groups, including nongovernmental organizations, can close the gap of singularity in interventions and maximize community reach. Sustainability and participation should drive advocacy plans. Media and communication channels are essential to promote the best practices of health. The engagement of AMR as a continued global health priority is paramount.

## 5 CONCLUSION

AMR control post‐COVID in Africa requires measures for continued antimicrobial stewardship and maintenance of good infection control practices. Robust surveillance of antimicrobial usage and resistance development is also pivotal. Deterring AMR development in Africa will thrive with more investment in implementing NAPs for AMR and demonstrating their sustained relevance. A policy framework that effectively describes the population's behavioral compass and interactions, the social and system entities that impact antimicrobial behavior, and suitable implementation design with appropriate risk communication, assessment, and regulation, establishes a strong focus for AMR management.

## AUTHOR CONTRIBUTIONS

Deborah O. Shomuyiwa, Don E. Lucero‐Prisno, Emery Manirambona conceptualization, project administration and design. Deborah O. Shomuyiwa, Don E. Lucero‐PrisnoIII, Emery Manirambona, Mohamed H. Suleman, Rehab A. Rayan, Junjie Huang, Thaint N. Zaw, Yusuf Babatunde, Salomey A. Denkyira, Shuaibu S. Musa data collection and literature review, preparation of the original draft and visualization. Deborah O. Shomuyiwa, Don E. Lucero‐Prisno, Emery Manirambona supervision, writing, reviewing, editing and proofreading. All authors have read, and confirm that they meet ICMJE criteria for authorship.

## CONFLICT OF INTEREST

The authors declare no conflict of interest.

## TRANSPARENCY STATEMENT

Deborah Oluwaseun Shomuyiwa affirms that this manuscript is an honest, accurate, and transparent account of the study being reported; that no important aspects of the study have been omitted; and that any discrepancies from the study as planned (and, if relevant, registered) have been explained.

## Data Availability

Data sharing not applicable to this article as no datasets were generated or analyzed during the current study.
